# Feasibility and acceptability of delivering adolescent health interventions alongside HPV vaccination in Tanzania

**DOI:** 10.1093/heapol/czv119

**Published:** 2016-01-14

**Authors:** Deborah Watson-Jones, Shelley Lees, Joseph Mwanga, Nyasule Neke, John Changalucha, Nathalie Broutet, Ibrahim Maduhu, Saidi Kapiga, Venkatraman Chandra-Mouli, Paul Bloem, David A Ross

**Affiliations:** ^1^Faculty of Infectious and Tropical Diseases, London School of Hygiene & Tropical Medicine, Keppel Street, London, WC1E 7HT, UK,; ^2^Mwanza Intervention Trials Unit, National Institute for Medical Research, Mwanza, P O Box 11936, Tanzania,; ^3^Faculty of Public Health and Policy, London School of Hygiene & Tropical Medicine, Keppel Street, London, WC1E 7HT, UK,; ^4^National Institute for Medical Research, Mwanza, P O Box 1462, Tanzania,; ^5^World Health Organization, Geneva, Switzerland,; ^6^Immunization and Vaccine Development, Ministry of Health & Social Welfare, Dar es Salaam, Tanzania and; ^7^MRC Tropical Epidemiology Group, Faculty of Epidemiology and Public Health, London School of Hygiene & Tropical Medicine, Keppel Street, London, WC1E 7HT, UK

**Keywords:** Adolescent, health, HPV, integration, Tanzania, vaccine

## Abstract

**Background:** Human papillomavirus (HPV) vaccination offers an opportunity to strengthen provision of adolescent health interventions (AHI). We explored the feasibility of integrating other AHI with HPV vaccination in Tanzania.

**Methods:** A desk review of 39 policy documents was preceded by a stakeholder meeting with 38 policy makers and partners. Eighteen key informant interviews (KIIs) with health and education policy makers and district officials were conducted to further explore perceptions of current programs, priorities and AHI that might be suitable for integration with HPV vaccination.

**Results:** Fourteen school health interventions (SHI) or AHI are currently being implemented by the Government of Tanzania. Most are delivered as vertical programmes. Coverage of current programs is not universal, and is limited by financial, human resource and logistic constraints. Limited community engagement, rumours, and lack of strategic advocacy has affected uptake of some interventions, e.g. tetanus toxoid (TT) immunization. Stakeholder and KI perceptions and opinions were limited by a lack of experience with integrated delivery and AHI that were outside an individual’s area of expertise and experience. Deworming and educational sessions including reproductive health education were the most frequently mentioned interventions that respondents considered suitable for integrated delivery with HPV vaccine.

**Conclusions:** Given programme constraints, limited experience with integrated delivery and concern about real or perceived side-effects being attributed to the vaccine, it will be very important to pilot-test integration of AHI/SHI with HPV vaccination. Selected interventions will need to be simple and quick to deliver since health workers are likely to face significant logistic and time constraints during vaccination visits.

## Introduction

At 1.2 billion, adolescents (10- to 19-year-olds) now make up one sixth of the world’s population (Sawyer *et al.* 2012), with almost 90% living in low and middle-income countries. Preventive interventions in this age group are often critical for future adult health and for the health of the next generation, in addition to adolescents’ own health (Goodburn and Ross 2000).

One intervention is human papillomavirus (HPV) vaccination to prevent cervical cancer and genital warts. An HPV vaccination programme targeted to adolescent girls provides a potential opportunity to introduce or strengthen the delivery of other adolescent health interventions (AHI) since this age group tends to have fewer contacts with health services than younger children and older adolescents (Britto *et al.* 2001; Barker *et al.* 2005; Mmari *et al.* 2010; Nordin *et al.* 2010). Because HPV vaccines are targeted at girls who are not routinely receiving other vaccines or other public health interventions, establishing an HPV vaccination programme often requires special systems for social mobilization, cold chain and other logistics to establish school-based or outreach delivery (Wigle et al. 2013). Integrating AHI with HPV vaccination may therefore represent an efficiency saving, making programmes more sustainable within the context of a comprehensive approach to cervical cancer prevention and control as developed by WHO (WHO 2013a, 2014a; Broutet *et al.* 2013).

Initial indications are that HPV vaccination can be delivered with high coverage in the target populations in sub-Saharan Africa (LaMontagne *et al.* 2011; Binagwaho *et al.* 2012; Watson-Jones *et al.* 2012a; Ladner *et al.* 2014). GAVI is now supporting demonstration projects and national programmes for HPV vaccination in low income countries (LICs) (GAVIAlliance 2012). As part of demonstration project support, countries should explore the possibilities of integrating other AHI alongside HPV vaccination. The Tanzanian Ministry of Health and Social Welfare (MoHSW) is now conducting a GAVI-supported demonstration project in Kilimanjaro Region aimed at targeting girls enrolled in primary school class (standard) 4 and out-of-school girls aged 9 years, prior to a national HPV vaccination programme. We conducted this study to assess the feasibility of integrating AHI with HPV vaccination. We explored which AHI and school health interventions (SHI) are currently recommended and being successfully implemented in Tanzania and identified a potential set of health interventions that could be delivered with the HPV vaccine within Tanzania.

## Methods

There were three project components. A stakeholder meeting was conducted in Dar es Salaam in May 2013 with policy makers and partners. Participants at the stakeholder meeting included clinical and non-clinical scientists with experience in research on cervical cancer prevention and adolescent health from the National Institute for Medical Research and LSHTM in Tanzania; senior clinicians working in cervical cancer from the MoHSW Reproductive and Child Health Unit (RCHU) and Ocean Road Cancer Institute; senior representatives from the School Health Programme (SHP) and Immunisation and Vaccine Development (IVD) Programme, the Tanzania Commission for AIDS, the Ministry of Education and Vocational Training (MoEVT), UNICEF, UNAIDS, the Prime Minister’s office, and WHO. Representatives from the Centers for Disease Control, Jhpiego, the Medical Womens Association of Tanzania, the International Centre for Reproductive Health, the Tanzania National Nursing Association and Marie Stopes International (MST) who were working in cervical cancer prevention also attended. Following summaries on the rationale for integrating AHI and HPV vaccine delivery, potential AHI and evidence about their potential effectiveness, and the design of the HPV vaccination demonstration project, recommendations were sought for a potential package of AHI/SHI suitable for delivery within an HPV vaccination programme through discussions and group work. Participants were allocated to four groups to discuss: (a) which four additional interventions should be prioritized, (b) whether boys, as well as girls, should be targeted for any of these interventions, and (c) whether any of the selected interventions should target children who were enrolled in school standards other than standard 4. To ensure transparency and to give equal weight to each participant’s views, after brainstorming and discussion of each issue, each participant was allocated the same number of votes. The group’s decision on prioritizing options for each question was based on total votes for each option.

Key informant interviews (KIIs) were conducted in July and August 2013 with national policy makers and programme managers. Key informants (KIs) included individuals working in cervical cancer prevention, treatment and control, vaccination services and adolescent health and school health programming. Eighteen KIs were interviewed, including senior representatives from the MoHSW, the RCHU, the SHP, and IVD programme; the MoEVT and Ministry of Information, Youth, Culture and Sports (MIYCS), and district health officers and District SHP Coordinators from Mwanza city’s Misungwi and Nyamagana Districts. These districts were chosen because of prior involvement in an HPV vaccine demonstration project (Watson-Jones *et al.* 2012a). KIs were identified through the stakeholder workshop and at Technical Advisory Group meetings of the national Cervical Cancer Control Programme. They provided recorded verbal and written consent including permission to record the interviews and were interviewed using semi-structured interview (SSI) guides (in English for KIs based in Dar es Salaam and in Swahili for Mwanza-based KIs). These listed topics and issues to be covered during interviews. KIs were asked about cervical cancer prevention, other AHI/SHI, facilitators and challenges to implementing these and which potential interventions might be suitable for integration with HPV vaccination. Digital recordings were transcribed and Swahili interviews were translated into English. The transcripts were imported into NVIVO version 9 (QSR International). A framework analysis approach was used to code themes guided by the research questions. Themes included knowledge and experiences of current vaccine programmes, knowledge about cervical cancer and the HPV vaccine, current SHI, views on integrating HPV vaccination with a health intervention, and potential challenges to integration.

A desk review conducted concurrently by one Tanzanian researcher was designed to identify and review key policies, guidelines and programme reports related to health services provided to adolescents in Tanzania, with a particular focus on those delivered in government primary schools. Potentially relevant documents were identified through a brainstorming exercise by the researchers, from relevant ministry, government, development partner and non-governmental organization websites and from KI suggestions. Available reports and guidelines of existing AHI/SHI, vaccination annual reports and annual district reports were requested as necessary. A total of 39 documents were reviewed, including reports on school health interventions (*n* = 5), MoHSW policies (*n* = 14), MoEVT policies (*n* = 5), policies from other sectors related to SHI and immunization (*n* = 13) and national guidelines on cervical cancer prevention and control (*n* = 2). Ten separate documents, primarily guidelines, were used to verify specific statements and actions in the policies reviewed. These guidelines had been mentioned in policy documents and, where the guideline existed, these were included in the review.

Documents were systematically reviewed using a pre-prepared data collection form and then coded and analysed manually using themes derived from the study objectives as described for the KII analysis.

## Results

Results are presented by pre-identified themes.

### HPV vaccination experience and views on school-based vaccination

Previous experiences with HPV vaccination in Tanzania were reviewed at the stakeholder meeting to set the context of the discussions (Watson-Jones *et al.* 2012a; Remes *et al.* 2012). The pilot HPV vaccination project conducted in Mwanza between 2010 and 2011 showed that school vaccination teams could be small (one, or occasionally two, nurses) who would normally only visit a particular school on one day during each vaccine dosing round (Watson-Jones *et al.* 2012a). For school-based health activities, nurses generally reach schools using public transport or on foot. On HPV vaccination days their responsibilities included packing vaccine, liaising with teachers, setting up immunization stations, providing information to pupils, performing vaccinations, completing vaccine registers, cards and tally sheets, and returning the vaccines to the health facility. At the start of the Mwanza project, communities, health workers and teachers recalled previous negative media coverage about the deworming programme, where pupils experienced side effects related to praziquantel which impacted TT vaccine uptake because community members believed that the vaccine had caused these side effects (Mwandoloma 2008). Having a non-positive opinion about the deworming programme was associated with failure to receive HPV vaccine in the Mwanza project (Watson-Jones *et al.* 2012b).

Most stakeholders and KIs were positive about offering HPV vaccine in schools, which was seen to be the most cost-effective and manageable approach to reach girls. Only one KI, from the RCHU, had concerns about school-based delivery because she believed it contradicted school health policies that stated that service provision should be conducted at health facilities with the school role restricted to providing linkages to health facilities.

### Current adolescent and school health interventions

The MoEVT and MoHSW, through the SHP, are responsible for coordinating and delivering SHI. Of the 14 SHI implemented in Tanzania at the time of the study, three were education-based, two were immunization-based and the remaining eight focused on Neglected Tropical Diseases, school health assessments (SHA) and nutrition (Table 1). Four SHI (sexual and reproductive health, hygiene and sanitation education in science classes, physical education, life skills and counselling training) were nested within the school teaching curriculum while the other 10 SHI were non-curriculum-based, with visits from health workers or the use of media for various activities or general school environment or feeding initiatives. Although little information in the form of reports on delivery of current programmes were available, it appeared from the data collection and KII that not all interventions were being implemented throughout the country. In 2013, the school-feeding programme, for example, operated in only 13 of the 169 districts and was scattered across five of the 30 regions of Tanzania, whilst the counselling intervention was only functioning in 4 of the 30 regions. The deworming and the school WASH programmes were also being conducted in selected districts.

**Table 1.** Current School Health Interventions in Tanzania.

Curriculum-based interventions				
Name of the Intervention		Target group	Mode of delivery	Coverage
Science (includes sexual and reproductive health, hygiene and sanitation,)		7–14 years (STD I–VII) targeting both girls and boys	Delivered by trained teachers (3–4 classes per week)	Nationwide
Physical Education and Personality		7–14 years (STD I–VII) targeting both girls and boys	Delivered by trained teachers (3 classes per week)	Nationwide
Life Skills		7–14 years (STD I–VII) targeting both girls and boys	Delivered by trained teachers (3–4 classes per week)	Nationwide
Guidance and Counselling		7–14 years (STD I–VII) targeting both girls and boys	Delivered by trained teachers using guidelines prepared by the MoEVT, MoHSW and other relevant stakeholders	Tanga, Lindi, Mtwara, Mbeya, Iringa, Ruvuma, Morogoro and Kilimanjaro.
Non-curriculum-based interventions				
Type of intervention	Target group	Mode of delivery		Coverage
BCG Vaccination	Targets girls and boys aged <7 years	During school enrolment teachers should check whether children have been immunized with BCG. If a child is suspected of not receiving this, the child is then referred to a health facility to be immunized		Nationwide
Tetanus Toxoid (TT) Vaccination	Target age: 15 years (STD VI–VII)	Teachers should check girls’ immunization cards and refer any girl who has not been immunized with TT to a health facility for immunization		Nationwide
Student Health Assessment (SHA)	Pre-primary (5–6 years) targeting girlsand boys Primary school girls and boys	Health workers are supposed to visit every primary school annually to conduct student health assessments e.g. vision checks, height and weight, blood pressure checks		Nationwide
First Aid Services	7–14 years (STD I–VII) targeting boys and girls	Health workers should provide first aid (usually in their health facility) when there is an emergency		Nationwide
Schoolgirls’ Pregnancy Check	Older pupils (STD V–VII) targeting girls of reproductive age	If a teacher suspects that a girl in the school is pregnant, all girls of reproductive age should be screened for pregnancy. A female teacher (or the school matron if there is one) performs the initial check by conducting a pelvic abdominal palpation and those suspected of being pregnant are sent to the nearest health facility for a pregnancy test		Nationwide
School Environment (includes WASH programme)	7–14 years (STD I–VII) targeting boys and girls	Teachers should supervise the students to keep the school rooms and grounds clean and tidy with community involvement of latrine construction, tree planting to provide shade and construction of wells to provide clean water		Nationwide
School Feeding	Primary (7–14 years) and Pre-primary (5–6 years)	School workers are supposed to prepare food for the students. Parents and community should contribute food supplies		Arusha, Manyara, Shinyanga, Dodoma and Singida
Radio or TV Programmes in Schools	7–14 years (STD I–VII) targeting boys and girls	Artists and media staff from the Ministry of Information, Youth, Culture and Sports (MIYCS), collaborating with the MOEVT and NGOs, are supposed to make occasional visits to schools to promote sport		Nationwide
Deworming for Schistosomiasis using Praziquantel	7–14 years (STD I–VII) targeting boys and girls	Delivered via a campaign basis once per year in regions where the Schistosomiasis prevalence is considered high enough to justify mass treatment. Trained teachers give the drug to the students under the supervision of health workers		Mainly in regions with high prevalence
Deworming for Soil Transmitted Helminths (STH) using Albendazole/Mebendazole; for Lymphatic Filariasis and Oncocerciasis using Mectizan; Trachoma using Zithromax	Targets the whole community including primary and secondary school students aged 7–20 years	The deworming campaign is conducted annually as part of the Neglected Tropical Disease Control Programme (NTD), using a community-based approach. Health workers and/or trained community drug distributors visit distribution venues and/or make house-to-house visits to administer medications to all students and other community members within the target age range		Mainly in regions with high prevalence

The only national experience of adolescent and school-based vaccination was from the tetanus toxoid (TT) vaccination programme which offered TT to 15 year-old girls as well as pregnant women. Challenges faced by this programme were felt to provide useful lessons for a future HPV vaccination programme, including rumours that the TT vaccine caused infertility because it was only being given to girls: ‘*there are rumours that when you vaccinate the girls they just are unable to have a baby or become infertile. So if we are targeting all girls we must expect these rumours*’ (Cervical Cancer focal person). Additional challenges included accessing girls who were absent from school or who had dropped out of school and it was suggested that, for HPV vaccination, out-of-school girls could be reached through African Child Health Days, (which conduct health promotion activities and intervention deliveries to children and adolescents), and other immunization campaigns. It was emphasized that girls should be vaccinated as early as possible: ‘*Truancy begins when the girls reach Standard four or Standard five, usually to earn money for the family*’ (Acting District Education Officer).

### Perceptions of SHP/AHI delivery

From the desk review, issues related to the SHP that were relevant to integrating HPV vaccination alongside this programme included vertical delivery mechanisms for different interventions, variable coverage of different SHI, financial and human resource limitations, coordination of a large number of collaborating actors and partners, different funding sources for different interventions, and lack of advocacy.

There were different perceptions about the relationships between the MoHSW and the MoEVT: ‘*We are seeing these challenges of poor collaboration, poor coordination and we are seeing that things are not moving without a team spirit…*’ (senior MoEVT staff member). In contrast a MoHSW representative stated ‘… *we have health and education counterparts in every region and every district … teachers are implementing a lot of the health interventions, … so our relationship is positive although we need to maintain and to strengthen it*.’ This view was echoed at district level: ‘*We, as the health department, are very happy when we see the teacher teaching the student about reproductive health education; he/she is teaching the student about diseases which can be prevented by being vaccinated, you see.*’ (District Medical Officer).

There was general recognition that the education and health sectors should work together to deliver the SHP since it was appreciated that poor health can impact a child’s education: *‘…they should cooperate because the students’ health also raises the standard of education to a big extent including their academic ability. Because if someone is sick s/he can’t attend, s/he can’t think well, s/he can’t read well.’* (District SHP Coordinator).

No reports provided objective data as to how well interventions were implemented and what impacts they might have had. Activities were described as being delivered vertically and only a few individuals participating in the stakeholder meeting or KII had been directly involved in the SHP. This meant that SHP interventions were often not familiar to many stakeholders and KIs. They could generally describe what should happen for some interventions but often did not seem to know, or were less forthcoming about, how the interventions were actually being delivered or whether SHP/AHI interventions were being delivered effectively. For example, few appeared to realize that mass treatment for schistosomiasis was currently only being offered in some regions and that mass treatment for soil-transmitted helminths was being delivered by community drug distributors as well as by health workers. Only one KI (from the SHP) admitted that a number of SHP interventions were being implemented with incomplete coverage and had failed to meet targets: *‘…* *there should be one latrine hole for twenty girls and one latrine hole for twenty five boys. The current average is one hole for fifty five boys and one hole for fifty seven girls … For school deworming, it started massively around 2000, and it is supposed to be done every year but, because of the huge resources needed, it has been done by phases incorporating several regions per year.’*

Deworming was reported to be a ‘successful’ health intervention in schools because the intervention was simple to administer with the help of trained school teachers, with one senior health official stating ‘*It is doing well maybe because they are pills, not injectables*’. Other SHI that were reported to be successful by several KIs included sanitation initiatives, such as latrine construction, and curriculum-based educational sessions (e.g. family planning, condom education, life skills education and the MEMA kwa Vijana adolescent sexual and reproductive health intervention in Mwanza Region (Ross *et al.* 2007)). However, with the exception of curriculum-based initiatives, where it was felt that intervention delivery had to strictly follow the curriculum, it was often difficult to elicit specific reasons about why interventions were considered ‘successful’. District health officials, who often have to do or supervise actual implementation, provided anecdotal evidence about which interventions were being delivered well, but most of these were not backed up by objective data. For example, a District School Health Coordinator attributed a fall in pregnancies to the SHP without specific evidence of this.

### Previous experience of integrated programmes for SHP/AHI

Several policy documents advocated for integrated implementation of health interventions (Ministry of Health and Social Welfare 1998, 2011a,b). Although there has been some integration of services within the health and education sectors, including provision of youth-friendly services through the maternal and neonatal health services (Ministry of Health and Social Welfare) , the desk review revealed only one example of an integrated package of SHI. This was funded by the Schistosomiasis Control Initiative (SCI) in 2008 and involved 452 schools in 34 districts in 6 regions (Ministry of Health and Social Welfare). Praziquantel was delivered to pupils aged 7–14 years old with other interventions including measles vaccination and mebendazole for in-school and pre-school children. Reported challenges included limited provision of information to the community, community misconceptions about the interventions (e.g. measles vaccine being seen as a cure for measles infection), problems with intervention delivery during examinations, health worker concerns about teachers dispensing medications, underestimation of class sizes resulting in stock-outs, delays in getting supplies to remote districts, and a lack of record-keeping and documentation on how logistical issues were handled.

### Interventions and target groups for inclusion in an integrated package

The stakeholder groups discussed potential AHI, including those found to have had beneficial outcomes in a recently published review (Box 1) (Broutet *et al.* 2013; Hindin *et al.* 2014). It was generally agreed that it would be desirable to include interventions for boys in any intervention package, and that interventions should not necessarily be limited to the school class or age group receiving the vaccine. Targeting some interventions to boys and also to girls outside standard 4, the target group for HPV vaccination, was considered important by a number of senior KIs: *‘It would be quite silly if you only give these additional treatments to those who are being provided HPV [vaccine] and leave the ones who are not targeted with HPV vaccine when you know that the other children also need [the interventions].’* (Senior MoHSW staff member).

**Box 1.** Adolescent health interventions discussed at stakeholder meeting

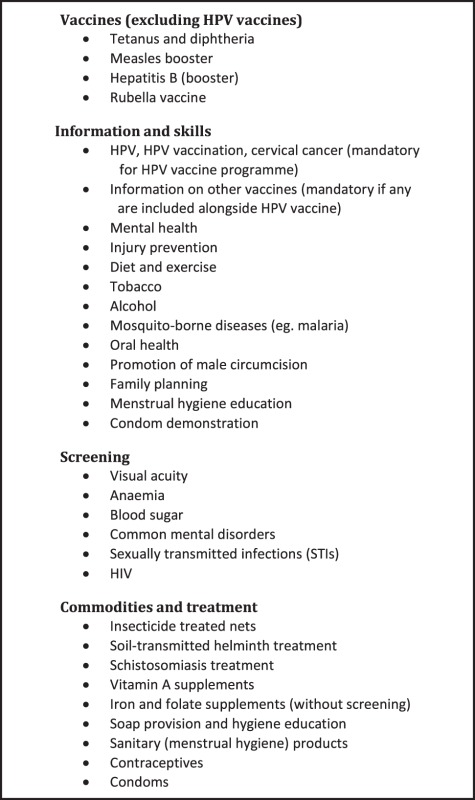										

However, there was no consensus between the groups in terms of what the priority interventions should be for inclusion in an HPV vaccination programme. The two most popular interventions mentioned by stakeholder meeting groups were deworming and vision screening, followed by life style education, oral hygiene education, with or without provision of a toothbrush and toothpaste; diet and exercise education; and sexual and reproductive health (SRH) education (Table 2).

**Table 2.** Overall scores by stakeholder groups for priority interventions by type.

Type of intervention	Specific intervention	Votes	% of total votes
Other vaccines	None specified by any group	0	0
Information & skills	Lifestyle education	20	10
	Oral health education (with or without provision of toothbrush and paste)	18	9
	Diet & exercise education	17	8
	Sexual & reproductive health and rights	12	6
	Hygiene education (with or without provision of soap)	12	6
	Health promotion through the school health programme	12	6
	Road safety education	10	5
	Education on mental health	8	4
Screening	Visual acuity	31	15
	General health checks	15	7
Commodities & treatment	Deworming, including schistosomiasis and/or soil-transmitted helminth treatment	38	18
	Youth-friendly services provision	12	6
	Provision of school meals	3	1
Total		208	100

In both the stakeholder meeting and the KII, very little evidence was provided by participants for their choice of potential interventions. There was a general consensus by KIs that an integrated adolescent health package would be an effective use of resources. Interventions mentioned as being suitable for integration again included deworming; vitamin A supplements (only one KI stressed that vitamin A supplements are usually targeted to under 5 year olds and that there is no current policy for these to be given to school-age children); health education, such as on menstrual hygiene, or condom demonstrations (although there are restrictions on these being permitted in schools); supplements including iron tablets and folic acid; vision screening; TT vaccination; and HIV and family planning education. There was less support for bednets, hand washing and physical exercise interventions. One KI (MoHSW staff member) stated *‘…some of the information things are very good, like (the importance of) physical activity, soap provision and hand washing, that can be delivered to all ages, but …, if you integrate it, it takes more time. I don’t think the staff we have are able to manage that. Things like soap, they can be done by anyone in schools, … it doesn’t need the health provider to go to school to teach children about hand washing. Things like hand washing can be incorporated into school programs. Physical exercise should be part of school programs and part of school health education. And I don’t think we should add this load to our few health workers to do something like that*.’

Generally stakeholders felt that HPV vaccination should be linked to an existing effective health programme, rather than to a new intervention. Since the school curricula already contained educational information on anaemia, nutrition and sexual and reproductive health, some participants stated that there might be limited benefit in repeating some of this information. Stakeholders also mentioned concerns that insecticide-treated nets (ITNs) might be misused as fishing nets, and that anaemia screening with finger-prick blood testing might reduce attendance at subsequent vaccination rounds. Some participants also stated that, since annual health screening in schools is not universal, HPV vaccination should not be linked to this activity.

### Challenges to integrating SHI with HPV vaccination

Challenges to integration mentioned at the stakeholder meeting included transport of health workers to schools and how they would carry additional items, such as bednets, in addition to vaccine, and whether nurses would actually have time to deliver additional interventions on HPV vaccination days. ‘*Education on menstrual hygiene, for example, that could be appropriate because we have early puberty and sexual maturation in young girls these days … However, does the health worker have the time to deliver those interventions’?* (Senior MoHSW staff member).

There was also a perception among a few KIs that additional funding to cover logistics, training and delivery would be needed to enable additional interventions to be delivered since coverage for many SHI is currently very low.

Finally, the impact on health facilities when health workers carry out additional outreach work was raised by a number of KIs including a senior MoHSW representative: *‘…* *we have a workforce shortage of 48%. … the same health worker, who is based at the dispensary, is also supposed to provide other health services, [but] we want to ask that same person to go out to conduct outreach services.’*

Challenges related to teacher involvement and their coordination with health programmes to implement an in-school HPV vaccination were not mentioned during the stakeholder meeting or KII. Although teachers provide education on health promotion and therefore reinforcing the importance of HPV vaccination may need to get incorporated into these messages, general discussions on the challenges of integrating HPV vaccination with other AHI focused on the health worker perspective, However one KI (a senior MoEVT representative) did mention that teacher involvement could be a facilitator to the interventions being accepted by parents: ‘*If daughters of the teachers will not get this vaccination then it will be a challenge for other learners. So it is important the teachers take it first because…….if a teacher, the head of school, the district education officer stands for it…school matron, school patron if they stand for it and support it then it will go as planned.’*

## Discussion

We explored the feasibility of integrating an adolescent health package with HPV vaccination in Tanzania through a 3 step process comprising a desk review, a national stakeholder meeting, and KII.

There was general support for the concept of integrating SHI/AHI with HPV vaccine, similar to findings from a South African study (MacPhail *et al.* 2013). Whilst the policy environment in Tanzania for the integration is also encouraging, actual experience of integrating health interventions, especially among adolescents, is very limited and the one SHI programme that attempted doing this at scale faced several obstacles. In addition, the country has only limited vaccination experience in schools through TT vaccine campaigns which met with major challenges. These findings emphasize the importance of careful planning, communication and involvement of all the important stakeholders, including health workers, teachers and their supervisors, religious leaders, politicians, parents and the students themselves. There may be added benefits for integrating HPV vaccine with other initiatives. Planning for this could act as a stimulus for a much better exchange of information between key stakeholders about what is being done, implementation lessons, and the creation of a platform for continued exchange and joint planning. Mozambique provides one example of setting up systems to facilitate inter-sectoral collaboration which could be applied to integration of HPV vaccination and other AHI. Inter-sectoral coordination committees involving the health, education and youth ministries were set up at national, provincial and district levels. These committees were mandated and supported to bring together the three sets of activities into one coherent whole (Chandra-Mouli *et al.* 2015).

Suggestions for which interventions to integrate with HPV vaccine during the stakeholder meeting and KIIs tended to focus on interventions that are relatively widely implemented already. Deworming was the most frequently mentioned, and there was also some support for health workers providing health promotion messages including SRH education. The latter may prove to be acceptable to parents since demand for educational messages on SRH from parents was also reported in the South African study mentioned above (MacPhail *et al.* 2013). There was widespread support that additional interventions should involve boys and girls beyond the target ages for vaccination, when appropriate. In our study there were mixed views on the importance and feasibility of delivering interventions such as vitamin A supplements, TT vaccine and vision checks alongside HPV vaccine.

Although KIIs gave an opportunity for individuals to discuss issues with an interviewer rather than in front of colleagues at a large stakeholder meeting, we found that little additional information or new ideas to inform the study objectives were gleaned through our KIIs. Overall, many stakeholders and KIIs had limited insight into current AHI/SHP activities and little awareness of how well these were performing. Specific suggestions for an adolescent health package were therefore not based on evidence of effectiveness in this setting and some that were suggested were actually not appropriate because they targeted the wrong age group of adolescents or pre-school children. This meant that discussions on integrating specific interventions were often relatively non-specific with no general consensus on which other interventions to include, and active debate was difficult because many interventions lay outside stakeholders’ and KIs’ areas of expertise and experience. Often the respondents assumed that these programmes were performing better than the reality. Challenges to integrating AHI were rarely mentioned in discussions and interviews. In the stakeholder meeting, for example, no group mentioned that, if an injection or tablet had side effects, these could be blamed on HPV vaccination, as indeed happened with schistosomiasis treatment during the 2008 integrated SHI programme (Mwandoloma 2008).

A number of suggested interventions were not suitable for combining with school-based HPV vaccine delivery in Tanzania. For example, although vitamin A supplementation and provision of ITNs were often suggested, they are not currently targeted to school-age children and SHP KIs stated that the government is very unlikely to support their extension to this target group. Despite this, free provision of bednets was mentioned but, even if it was to be approved, there would be considerable logistical challenges for health workers to get these to schools, although this might be overcome by providing a voucher for a free net rather than the net itself. Visual acuity screening was also suggested by several KIs and also received support during the workshop, but there is evidence from studies in Tanzania that this is unlikely to be cost-effective (Ministry of Health and Social Welfare)^,^ and, although visual acuity screening can be done well by teachers who have been given only a brief training, the prevalence of visual acuity problems is relatively low in primary school children (Wedner et al. 2000). Furthermore, a study in secondary school students in Dar es Salaam showed that, even if students who screened positive were provided with transport costs and free visits to an optometrist, and were given free spectacles of a type chosen by the pupil, the great majority of pupils with significant visual acuity problems did not wear their spectacles to school because they were teased if they did (Wedner et al. 2000, 2002, 2008). HIV and family planning education, although sensitive, may be acceptable if offered to older primary school children outside the target class for the HPV vaccine in order to avoid a perception that there is a connection between the HPV vaccine and HIV or contraception. Hand-washing education interventions are relatively simple. A study in first year pupils in Chinese primary schools found that a single 40 min session taught by the class teacher and a gift of one bar of soap for the child to take home was associated with a borderline significant reduction in school absenteeism, and when this session and gift was supplemented with continuous provision of soap to the school, the reduction in absenteeism was statistically significant (Bowen *et al.* 2007). However, it is not known whether a single hygiene education session would impact illness and absenteeism rates in Tanzanian schools, and health education is already supposed to happen as part of the science course in schools, with practical exercises sometimes performed within school science clubs.

It may be appropriate to offer different interventions with each dose of HPV vaccine. Currently, the Tanzanian MoHSW will ask health workers to deliver two doses of HPV vaccine to each girl in primary school standard 4 following recent WHO recommendations for a two dose vaccination strategy (WHO 2014b), with one routine school visit for each dose, unless a substantial number of girls in a school miss the dose, where a second visit targeting these girls should be scheduled. Given the limitations of health worker time on vaccination days, a potential suitable package delivered in boys and girls in class 4 could comprise:

- health promotion messages including education related to sexual and reproductive health, and/or personal and oral hygiene at the first vaccination visit
- nutrition and exercise education, and/or TT vaccination and provision of other needed vaccines to classes other than class IV at the second vaccination visit.

The GAVI-funded demonstration programme in Tanzania provides an opportunity to further explore integration of HPV vaccination and other interventions. The explorative study described in this paper and the methods, tools and protocol developed for data collection have served as guidance for the development of a toolkit that other countries implementing HPV vaccine demonstration programmes are using (currently 23 HPV vaccination demonstration projects in low income countries are supported by GAVI) (WHO 2013b).

However, given the limitations of the methods identified by this work, including the lack of stakeholder familiarity of other programmes outside their area of expertise, for future planning on integrating services it would perhaps be more useful to identify a dedicated individual who is familiar with HPV vaccination delivery in order to explore with each programme what has been done and the lessons learnt, and to work through the potential benefits and negative consequences of adding an intervention alongside HPV vaccination with the specific programme leaders.

Given the financial, social and logistic constraints associated with SHI, and the limited experience with integrating different AHI, it will be important to test the integration of effective and acceptable, simple interventions with HPV vaccination to check that they do not add significantly to the time that the health worker is out of his/her station and that they do not affect HPV vaccine acceptability and coverage.

## Ethical considerations

The project received ethical approval from the Lake Zone Institutional Review Board, the Medical Research Coordinating Committee of the National Institute for Medical Research and the Ethics Committee of the London School of Hygiene and Tropical Medicine.

## References

[czv119-B1] Barker G Olukoya A Aggleton P. 2005 Young people, social support and help-seeking. International Journal of Adolescent Medicine and Health 17: 315–35. 16445071 10.1515/ijamh.2005.17.4.315

[czv119-B2] Binagwaho A Wagner CM Gatera M 2012 Achieving high coverage in Rwanda's national human papillomavirus vaccination programme. Bulletin of the World Health Organization 90: 623–8. 22893746 10.2471/BLT.11.097253 PMC3417784

[czv119-B3] Bowen A Ma H Ou J 2007 A cluster-randomized controlled trial evaluating the effect of a handwashing-promotion program in Chinese primary schools. American Journal of Tropical Medicine and Hygiene 76: 1166–73. 17556631

[czv119-B4] Britto MT Klostermann BK Bonny AE Altum SA Hornung RW. 2001 Impact of a school-based intervention on access to healthcare for underserved youth. Journal of Adolescent Health 29: 116–24. 11472870 10.1016/s1054-139x(01)00196-3

[czv119-B5] Broutet N Lehnertz N Mehl G 2013 Effective health interventions for adolescents that could be integrated with human papillomavirus vaccination programs. Journal of Adolescent Health 53: 6–13. 23643336 10.1016/j.jadohealth.2013.02.022

[czv119-B6] Chandra-Mouli V Gibbs S Badiani R Quinhas F Svanemyr J. 2015 Programa Geracao Biz, Mozambique: how did this adolescent health initiative grow from a pilot to a national programme, and what did it achieve? Reproductive Health 12: 12 25971669 10.1186/1742-4755-12-12 PMC4429477

[czv119-B7] GAVIAlliance. 2012 http://www.gavialliance.org/support/nvs/human-papillomavirus-vaccine-support/.

[czv119-B8] Goodburn EA Ross DA. 2000 Young people's health in developing countries: a neglected problem and opportunity. Health Policy Plan 15: 137–44. 10837036 10.1093/heapol/15.2.137

[czv119-B9] Hindin MJ Bloem P Ferguson J. 2014 Effective Nonvaccine Interventions to Be Considered Alongside Human Papilloma Virus Vaccine Delivery. Journal of Adolescent Health 2015; 56/1: 10–8. 25287988 10.1016/j.jadohealth.2014.08.004

[czv119-B10] Ladner J Besson MH Rodrigues M Audureau E Saba J. 2014 Performance of 21 HPV vaccination programs implemented in low and middle-income countries, 2009–2013. BMC Public Health 14: 670 24981818 10.1186/1471-2458-14-670 PMC4085395

[czv119-B11] LaMontagne D Barge S Le N 2011 Human papillomavirus vaccine delivery strategies that achieved high coverage in low- and middle-income countries. Bulletin of the World Health Organization 89: 821–30B. doi:10.2471/BLT.11.089862. 22084528 10.2471/BLT.11.089862 PMC3209730

[czv119-B12] MacPhail C Venables E Rees H Delany-Moretlwe S. 2013 Using HPV vaccination for promotion of an adolescent package of care: opportunity and perspectives. BMC Public Health 13: 493 23692596 10.1186/1471-2458-13-493 PMC3681713

[czv119-B13] Ministry of Health and Social Welfare. 2008. National Road Map Strategic Plan to Accelerate Reduction of Maternal, Newborn and Child Death in Tanzania, 2008–2015. Ministry of Health & Social Welfare, Dar es Salaam.

[czv119-B14] Ministry of Health and Social Welfare. 2011. National Adolescent Reproductive Health Strategy 2011–2015. Ministry of Health & Social Welfare, Dar es Salaam.

[czv119-B15] Ministry of Health and Social Welfare. 2007. First round of Schistosomiasis drug distribution to pupils in primary schools conducted in 2007. Ministry of Health & Social Welfare, Dar es Salaam.

[czv119-B16] Ministry of Health and Social Welfare. 2009. Second Round of School Deworming Treatment conducted in August 2008 in 6 Phase II regions in Tanzania Mainland. 2009*.* Ministry of Health & Social Welfare, Dar es Salaam.

[czv119-B17] Ministry of Health and Social Welfare. 1998 Policy Guidelines on School Health Promotion in Tanzania Mainland*.* Ministry of Health and Social Welfare, Dar es Salaam.

[czv119-B18] Ministry of Health and Social Welfare. 2011a National Adolescent Reproductive Health Strategy 2011–2015; Reproductive and Child Health Section, Ministry of Health and Social Welfare, Dar es Salaam.

[czv119-B19] Ministry of Health and Social Welfare. 2011b National Cervical Cancer Prevention and Control Strategic Plan 2011–2015. Reproductive and Child Health Section, Ministry of Health and Social Welfare, Dar es Salaam.

[czv119-B20] Mmari KN Oseni O Fatusi AO. 2010 STI treatment-seeking behaviors among youth in Nigeria: are there gender differences? International Perspectives on Sexual and Reproductive Health 36: 72–9. 20663743 10.1363/ipsrh.36.072.10

[czv119-B21] Mwandoloma H 2008. Vaccination campaign ends amid reservations. The Guardian, Tues 2 Sept 2008.

[czv119-B22] Nordin JD Solberg LI Parker ED. 2010 Adolescent primary care visit patterns. Annals of Family Medicine 8: 511–6. 21060121 10.1370/afm.1188 PMC2975686

[czv119-B23] Remes P Selestine V Changalucha J 2012 A qualitative study of HPV vaccine acceptability among health workers, teachers, parents, female pupils, and religious leaders in northwest Tanzania. Vaccine 30: 5363–7. 22732428 10.1016/j.vaccine.2012.06.025 PMC3409375

[czv119-B24] Ross DA Changalucha J Obasi AI 2007 Biological and behavioural impact of an adolescent sexual health intervention in Tanzania: a community-randomized trial. Aids 21: 1943–55. 17721102 10.1097/QAD.0b013e3282ed3cf5

[czv119-B25] Sawyer SM Afifi RA Bearinger LH 2012 Adolescence: a foundation for future health. Lancet 379: 1630–40. 22538178 10.1016/S0140-6736(12)60072-5

[czv119-B26] Watson-Jones D Baisley K Ponsiano R 2012a Human papillomavirus vaccination in Tanzanian schoolgirls: cluster-randomized trial comparing 2 vaccine-delivery strategies. The Journal of Infectious Disease 206: 678–86. 10.1093/infdis/jis407 PMC3414230 22711908

[czv119-B27] Watson-Jones D Tomlin K Remes P 2012b Reasons for receiving or not receiving HPV vaccination in primary schoolgirls in Tanzania: a case control study. PLoS ONE 7: e45231 23115621 10.1371/journal.pone.0045231 PMC3480345

[czv119-B28] Wedner SH Ross DA Balira R Kaji L Foster A. 2000 Prevalence of eye diseases in primary school children in a rural area of Tanzania. British Journal of Ophthalmology 84: 1291–7. 11049957 10.1136/bjo.84.11.1291 PMC1723290

[czv119-B29] Wedner SH Ross DA Todd J 2002 Myopia in secondary school students in Mwanza City, Tanzania: the need for a national screening programme. British Journal of Ophthalmology 86: 1200–6. 12386067 10.1136/bjo.86.11.1200 PMC1771368

[czv119-B30] Wedner S Masanja H Bowman R 2008 Two strategies for correcting refractive errors in school students in Tanzania: randomised comparison, with implications for screening programmes. British Journal of Ophthalmology 92: 19–24. 18156372 10.1136/bjo.2007.119198

[czv119-B31] WHO. 2013 Comprehensive cervical cancer prevention and control – a healthier future for girls and women. Available at: http://www.who.int/reproductivehealth/publications/cancers/9789241505147/en/, accessed 7 October 2014.

[czv119-B32] WHO, UNICEF, WHO and UNFPA. Toolkit. The Assessment of Adolescent Health Interventions in GAVI HPV Vaccine Demonstration Programmes. Available at: http://www.who.int/immunization/diseases/hpv/toolkit_assessment_AH_interventions.pdf?ua=1, accessed 18 November 2014.

[czv119-B33] WHO. 2014a Options for linking health interventions for adolescents with HPV vaccination. Available at: http://www.who.int/immunization/diseases/hpv/linking_h_interventions/en/, accessed 29 November 2015.

[czv119-B34] WHO. 2014b World Health Organization. Meeting of the Strategic Advisory Group of Experts on immunization, April 2014 – conclusions and recommendations. Weekly Epidemiological Record 89: 221–36. 24864348

[czv119-B35] Wigle J Coast E Watson-Jones D. 2013 Human papillomavirus (HPV) vaccine implementation in low and middle-income countries (LMICs): health system experiences and prospects. Vaccine 31: 3811–7. 23777956 10.1016/j.vaccine.2013.06.016 PMC3763375

